# Targeting terminal pathway reduces brain complement activation, amyloid load and synapse loss, and improves cognition in a mouse model of dementia

**DOI:** 10.1002/alz.090692

**Published:** 2025-01-09

**Authors:** Wioleta M Zelek, Ryan J Bevan, Bryan Paul Morgan

**Affiliations:** ^1^ Cardiff University School of Medicine, Dementia Research Institute, Cardiff United Kingdom; ^2^ Cardiff University, Cardiff United Kingdom; ^3^ UK Dementia Research Institute at Cardiff University, Cardiff, South Glamorgan United Kingdom

## Abstract

**Background:**

Neuroinflammation is a critical factor of Alzheimer’s Disease (AD). Dysregulation of complement leads to excessive inflammation, direct damage to self‐cells and propagation of injury. This is likely of particular relevance in the brain where inflammation is poorly tolerated and brain cells are vulnerable to direct damage by complement. Membrane attack complex (MAC) is highly pro‐inflammatory product of the complement cascade killing cells by lysis and/or causing ‘bystander’ damage initiating NLRP3 inflammasome activation and provoking other immune damaging responses leading to death of the vulnerable nerve cells.

**Method:**

The role of MAC in AD was investigated in MAC‐deficient animals and by using a newly developed anti‐C7 monoclonal antibody (mAb) that efficiently inhibits formation of the MAC *in vitro* and *in vivo*. Impact of C7 deficiency on brain complement dysregulation, synapse loss, amyloid load and cognitive decline was examined by comparing APP^NL‐G‐F^ mice back‐crossed to C7 deficiency (APP^NL‐G‐F^xC7) with unmodified APP^NL‐G‐F^ mice. To assess the effect of therapeutic C7 blockade, unmodified APP^NL‐G‐F^ mice were treated systemically (for four weeks) with anti‐C7 mAb or control IgG and the same set of parameters of complement dysregulation, pathology and cognition measured.

**Result:**

C7 deficiency in App^NL−G−F^ mice reduced levels of complement activation markers, reduced amyloid load and increased synapse density with a commensurate improvement in cognitive test performance. Systemic treatment of App^NL−G−F^ mice with a blocking anti‐C7 mAb reduced brain levels of complement activation markers, amyloid load and increased neuronal spine density in treated mice in peri‐plaque areas when compared to controls. in App^NL−G−F^ mice. APP^NL‐G‐F^xC7 performed significanlty better in behavioural cognitive tests.

**Conclusion:**

We demonstrate that complement dysregulation occurs in brain in mouse models of AD. C7 deficiency reduced brain complement dysregulation, improved pathological parameters and cognitive function; systemic anti‐C7 therapy reduced complement dysregulation and protected from synapse loss in the model. Modification for brain delivery of the anti‐C7 mAb will enhance efficacy in the model. The findings highlight the potential for complement inhibition at the level of MAC as a therapy in AD.

